# What can mathematical models bring to the control of equine influenza?

**DOI:** 10.1111/evj.12104

**Published:** 2013-08-02

**Authors:** J M Daly, J R Newton, J L N Wood, A W Park

**Affiliations:** * School of Veterinary Medicine and Science, University of NottinghamSutton Bonington, UK; † Centre for Preventive Medicine, Animal Health TrustNewmarket, UK; ‡ Department of Veterinary Medicine, University of CambridgeUK; § Odum School of Ecology and Department of Infectious Diseases College of Veterinary Medicine, University of GeorgiaUSA

**Keywords:** horse, influenza, mathematical models, vaccination

## Abstract

Mathematical modelling of infectious disease is increasingly regarded as an important tool in the development of disease prevention and control measures. This article brings together key findings from various modelling studies conducted over the past 10 years that are of relevance to those on the front line of the battle against equine influenza.

The Summary is available in Chinese – see Supporting information.

## Introduction

Equine influenza is a highly contagious viral disease with typical clinical signs in fully susceptible animals of a deep, hacking cough and nasal discharge with fever. It is rarely fatal, usually resolving within 2–3 weeks. However, it has the potential to be highly disruptive to training and competition schedules. As a result, vaccination against equine influenza has been mandatory for racing Thoroughbreds in the UK since 1981, and major outbreaks have been few and far between since then. Similarly, the Fédération Equestre Internationale has set requirements for vaccination against equine influenza for horses competing nationally and internationally in sports under its jurisdiction such as jumping, dressage and eventing. The incursion of the virus into Australia in 2007 reminded us of the impact that influenza can have in an unvaccinated population of horses 1.

The influenza A virus genome consists of 8 RNA (rather than DNA) fragments encoding up to 12 different proteins only. In terms of controlling disease, the 2 proteins that project from the surface of the virus are most important; these are the haemagglutinin (HA) and neuraminidase (NA) proteins. The HA, which is most abundant, is responsible for allowing the virus to attach and gain entry to cells in the respiratory tract of the host. The NA, however, enables the release of newly synthesised virus by cleaving the bond between the HA and receptors on the host cell. Influenza A viruses are classified into subtypes on the basis of the reactivity of the HA and NA proteins. There is only one subtype of influenza currently circulating in horses – the H3N8 subtype. A representative of the H7N7 subtype is still included in some equine influenza vaccines but no new isolations of this subtype have been reported in the last quarter century 2.

The mathematical simulation models discussed here build on the pioneering statistical modelling studies conducted using field data in the UK (e.g. 3,5) and North America (e.g. 6–7), which demonstrated the importance of humoural immune responses to vaccination in providing protection against challenge. Further, the mathematical models have depended to some extent on data from experimental studies in the target species, some involving infectious challenge (see supplementary data in 8 for a summary). The approaches described below have thus demonstrated the integrative qualities that mathematical modelling can bring to interdisciplinary collaborations.

## A fundamental model of an equine influenza outbreak

**Figure 1:**
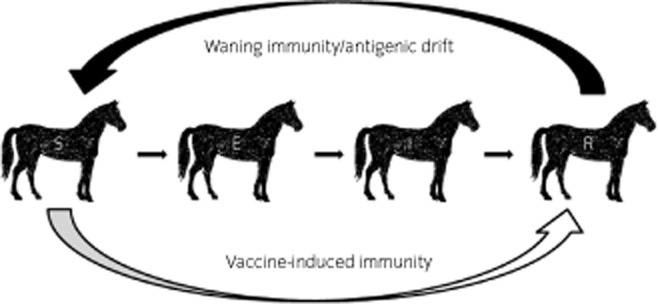
Diagram of the compartmental (SEIR) model. E = exposed; I = infectious; R = recovered; S = susceptible.

The basic features of an equine influenza outbreak can be captured using a relatively simple compartmental model (Fig 1; 9). The model can be used to simulate what happens if one infected individual is introduced into a closed population of, say, 100 individuals. At the start of an outbreak in a group of animals that have been neither vaccinated against nor previously infected with equine influenza, all 100 horses will be compartmentalised as being susceptible to infection (S). Whether or not any one susceptible horse becomes infected is a chance event. Hence, the model can incorporate such elements of chance. In a deterministic model, the model performs the same way for a given set of initial conditions, which in reality is unlikely to be the case. A stochastic model allows individual variation in the probability of, for example, a susceptible horse coming into contact with an infectious animal and that such a contact will result in transmission of virus to the susceptible individual. Essentially, the model is programmed to flip an appropriately weighted coin to determine whether an encounter between an infected and susceptible horse results in infection of the susceptible horse. For influenza, there is a period of around 2 days when an infected animal is not yet shedding virus (called the latent period). During this time, the horse is in the exposed (E) compartment. When an infected animal starts to shed virus, it enters the infectious (I) compartment. Finally, as influenza is not a chronic illness that results in prolonged subclinical infectivity (‘carrier’ state), a horse can be recovered from infection (R) and immune to infection. Waning immunity may eventually return the horse to the susceptible compartment. In a closed population, the infection is self-limiting; when contacts (which are assumed to be random) are unlikely to involve the susceptible–infectious transmission pair, then the outbreak stops. This can occur towards the beginning of the outbreak when infectious individuals are rare, or towards the end, when susceptible individuals are rare, and when previously infectious individuals have recovered and are resistant to re-infection.

**Figure 2:**
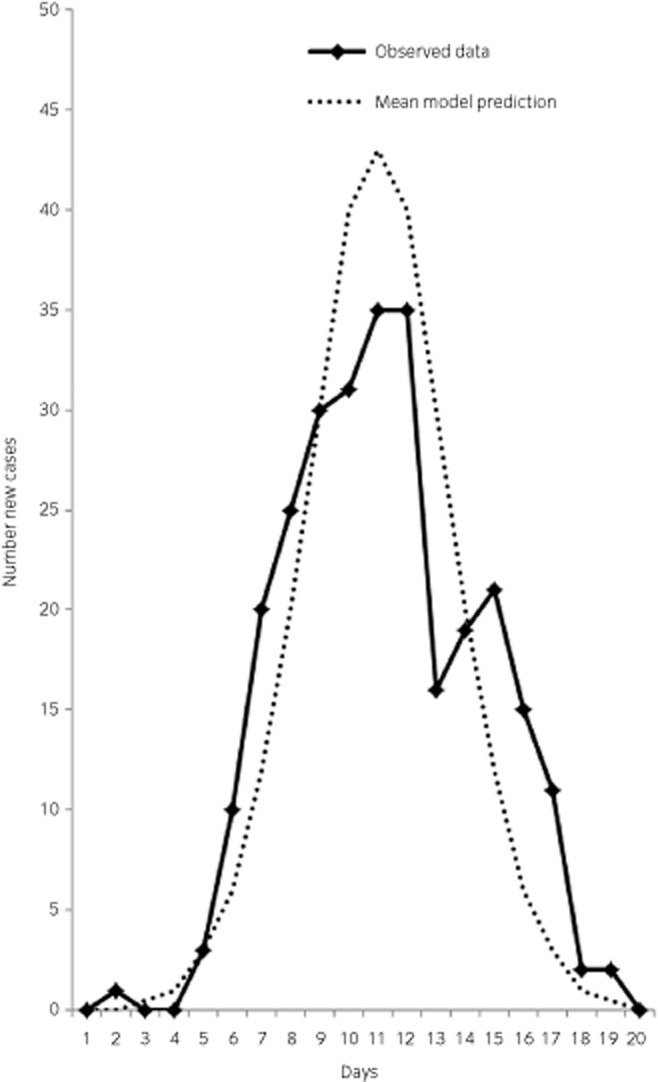
A simple stochastic compartmental (SEIR) model captures the features of an outbreak of equine influenza in a closed population of influenza-naïve horses on a single premise. The number of new cases arising each day as predicted by the model (dotted line) is superimposed over observed data from an outbreak in New York in 1963 (solid line). Redrawn from Glass *et al*. 10.

The output from one of the earliest versions of this model was derived from a naturally occurring outbreak of equine influenza in unvaccinated horses on a racetrack in the USA that occurred in 1963 and was validated as being accurate against a second outbreak in another susceptible population also in 1963 10 (Fig 2). This was when equine influenza of the currently circulating H3N8 subtype first emerged so the horses were almost certainly naïve to the virus. The model was used to estimate the basic reproduction number, R0 9. At a value of 10 (i.e. on average, introduction of one infected horse into a naïve population gives rise to 10 new cases), this is considered a high R0. Although this fits with the generally held perception that if equine influenza virus is introduced into a naïve population it can spread rapidly, a more conservative estimate (R0 = 2–5) was obtained using data from an outbreak of equine influenza in Japan 11, which is in better agreement with subsequent modelling studies 8. Estimation of R0 is challenging; results can vary according to methods used and the data available, which are influenced by factors such as population density and characteristics of the outbreak strain. Nonetheless, R0 is an important value as it reflects the feasibility of containing an outbreak, for example by allowing an estimation of what proportion of a population would need to be vaccinated to break the chain of transmission.

## Control of equine influenza by vaccination

The aim of vaccination is 2-fold: to protect the vaccinated individual from becoming ill and to limit the spread of infection. As a result of vaccination, horses are less likely to become infected, and hence infectious. In other words, vaccination can transfer a previously susceptible (S) animal into the recovered (R) and resistant to infection category (Fig 1), without going through the exposed (E) and infectious (I) categories; this reduces the proportion of susceptible individuals within the population and hence lowers the effective reproduction number (R). This is similar to the measure R0 but acknowledges that some individuals are fully or partially protected. Extending the basic model to take into account the effects of vaccination shows, as expected, that there is a dramatic reduction in the occurrence of outbreaks among groups of vaccinated horses 10–12. The model of Glass *et al*. (2002) also predicted that over 80% of outbreaks that do occur peter out with less than 5% of the population being infected 10. This suggested that many small outbreaks of equine influenza might go undetected in vaccinated populations, which was confirmed in subsequent surveillance studies (unpublished observations).

The effectiveness of vaccination primarily depends on the stimulation of circulating antibody to the HA protein to block attachment of the virus to host cells. Combining data from several experimental challenge studies involving a total of 32 unvaccinated and 57 vaccinated ponies demonstrated an empirical relationship between the levels of antibody measured by the single radial haemolysis (SRH) test and the probability of a pony becoming infectious when challenged with the same (homologous) strain as that contained in the vaccine used 13. The probability of becoming infectious is halved by vaccination. Vaccination also reduced the mean infectious period if a pony was infected (4.8 to 2.5 days) and extended the mean latent period (1.75 to 2 days).

Antibody levels stimulated by vaccination decline over time. Observational data combined from 618 racehorses were used to estimate the mean antibody level over the course of a year for horses vaccinated under the UK Jockey Club policy current at the time, i.e. an initial course of 3 doses with the first doses administered 21–92 days apart and the third dose administered 150–215 days after the second dose, followed thereafter by annual ‘booster’ doses 13. A model was developed informed by these data that incorporated realistic changes to the population structure of a typical UK Thoroughbred flat race training yard over the year, i.e. the loss of horses over 2 years old throughout July to December due to sales or injury and the introduction of yearlings peaking at the time of the October sales 13. The model illustrated that there are periods of high risk for epidemics occurring if equine influenza were to enter the yard 13. It was also shown that, in theory, reducing the interval between vaccinations for horses aged ≥2 years from 1 year to 6 months significantly reduces the risk that introducing an infected horse into a flat race training yard will result in an outbreak 13.

## Impact of antigenic drift on vaccine efficacy

**Figure 3:**
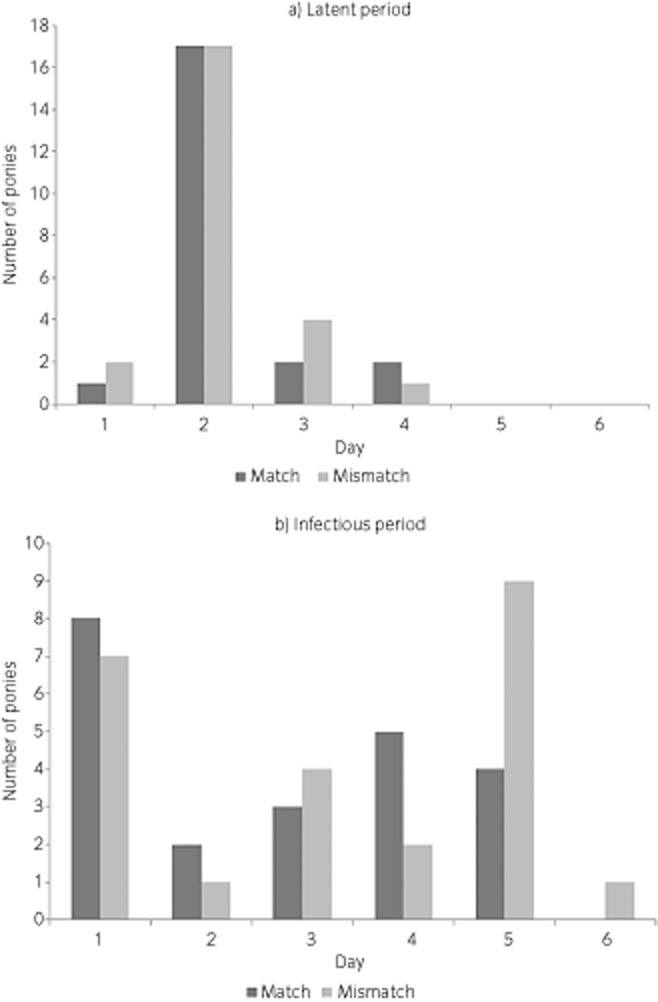
Empirical data on infectious period from vaccination and challenge studies of ponies receiving equine influenza vaccines containing strains that were homologous (matched) or heterologous (mismatched) to the challenge strain.

Equine influenza viruses are subject to a phenomenon known as antigenic drift, which results in evolution of viruses that are no longer fully recognised by antibodies generated by infection or vaccination with an earlier strain of the virus. This will result in animals that had been in the ‘recovered’ compartment being ‘susceptible’ to infection again (Fig 1). Models are only as good as the assumptions made in constructing them; the more real-life data that can be used to inform them, the better. For example, a horse does not immediately move from the ‘susceptible’ to ‘infectious’ compartments; similarly, the transition from ‘infectious’ to ‘recovered’ is not instantaneous. Some earlier infectious disease models made assumptions about the duration of the latent and infectious periods and assumed an exponential distribution of the transition rates between compartments. However, cross-protection experiments, in which ponies are exposed to virus and samples taken daily to measure virus shedding, allow accurate profiles to be obtained. There was no difference in the distribution of latent periods between ponies undergoing homologous (vaccine and challenge strain closely related) or heterologous (vaccine and challenge strain mismatched) challenge 14. However, the majority of ponies vaccinated with heterologous vaccine had a prolonged infectious period (5 days) whereas the majority of ponies vaccinated with a homologous vaccine only shed virus for one day, reducing the opportunity for spread of the virus (Fig 3). Therefore, although the use of out-dated strains in vaccines has relatively small effects at the level of the individual animal, mathematical models reveal that when scaled up to the population level they result in a significantly increased risk of an epidemic occurring 14.

To obtain antigenic data the virus must first be cultivated from live virus in swabs taken from infectious horses, whereas sequence data can be obtained rapidly and reliably even if sampling is not performed under optimal conditions. Therefore, the ultimate goal is to be able to link sequence data with the likelihood of vaccine failure. Mathematical modelling was used to relate the number of amino acid substitutions occurring in the HA protein to the likelihood of vaccine breakdown leading to larger outbreaks 8. This study demonstrated that with fewer than 2 amino acid changes in the HA sites targeted by antibodies between vaccine and infecting strain, transmission was limited. In contrast, large outbreaks are the most likely outcome if an infected horse is introduced into a population vaccinated with a vaccine containing an HA differing by 7 or more amino acids. However, it is not only the number but also the nature and location of amino acid changes that determine whether vaccine breakdown will occur 8.

## Understanding the evolution of equine influenza viruses

The H3N8 subtype viruses, which were first recognised in horses in the USA in 1963 15, initially evolved in a single lineage 16, as do human influenza A viruses. However, in the late 1980s, 2 distinct sub-lineages emerged that had apparently evolved independently on the American and Eurasian continents. In recent years, the picture has become more complex 17. Koelle *et al*. took a novel approach to model the different patterns of evolution observed for human influenza A viruses on one hand and human influenza B and equine influenza A in horses on the other 18. They modelled genetic evolution of the virus and the epidemiological dynamics separately and then combined the 2, which is computationally less demanding than simulating the whole process at once. Treating the American and European continents as a single continuum (a ‘patch’) allowed variants of equine influenza virus that arose in America to rapidly spread across both continents, replacing the previous variant and resulting in a single lineage. This was consistent with field observations in which the ‘patch’ continuum between Europe and America was effectively created through unrestricted transport by air of infectious horses between continents. Dividing the continents into 2 patches (corresponding to the introduction of quarantine in 1986) led to separate lineages evolving independently on the 2 continents with limited opportunities for exchange of antigenic variants. Relaxation of quarantine restrictions on the European side led to the introduction of the American variant into Europe and co-circulation of 2 lineages for some time as has been observed. An important concept illustrated by this modelling exercise was that the similar patterns of evolution observed for equine influenza A and human influenza B (i.e. the co-circulation of lineages) could have different underlying mechanisms. For human influenza B, 2 distinct lineages were generated by simulating a longer duration of infection, which is biologically plausible as influenza B primarily infects children and they have longer infectious periods than adults.

## Using models to assess outbreak control measures

The studies described thus far were restricted to studying what happens in a small, closed population, such as a horse-training yard of up to 100 animals. One of the potential advantages of using mathematical modelling is the ability to scale up *in silico* to a broader population in which it would be impossible to conduct experimental infection studies. However, there are many more variables in larger-scale outbreaks.

Mathematical models already demonstrated that vaccination (especially with vaccines containing up-to-date strains) can work at a within-yard level (Table 1). A meta-population model incorporating spread from yard-to-yard informed by data from the 2003 outbreak of equine influenza in Newmarket, which involved spread of infection between multiple yards 19, confirmed the previously anecdotal belief that vaccination in the face of an outbreak can be an effective control measure 20. The model also suggested that ‘poor responders’ can have a significant impact on the effectiveness of vaccination policies, particularly if these are clustered within a few yards in which there is active noncompliance with mandatory vaccination policies. This model made some assumptions, however. Firstly, it was assumed that the probability of contact was the same for all horses, whereas other authors have shown that, even in a population of horses housed and exercised in a relatively confined area, mixing is not homogeneous 21. Secondly, it was assumed that each individual responded to infection in the same way.

**Table 1:** Summary of key features of equine influenza described in mathematical models

Feature	Reference
Equine influenza is highly contagious	Glass *et al*. (2002) 10
Vaccination reduces the occurrence of epidemics	de la Rua-Domenech *et al*. (2000) 12; Glass *et al*. (2002) 10
The majority of outbreaks in a vaccinated group of horses are of limited size	Glass *et al*. (2002) 10
Strategic timing of vaccination can reduce the risk of outbreaks occurring when horses congregate for racing or sales	Park *et al*. (2003) 13
Although of little consequence at the individual level, a mismatched vaccine strain increases the likelihood of larger outbreaks occurring	Park *et al*. (2004) 14
Effective quarantine prevents incursion of novel equine influenza strains	Koelle *et al*. (2010) [18]
Vaccination in the face of an outbreak is an effective control measure	Baguelin *et al*. (2010) 20
Individuals within a group of vaccinated animals that remain unvaccinated or respond poorly to vaccination can have serious consequences	Baguelin *et al*. (2010) 20

The outbreak of equine influenza in the naïve equine population in Australia in 2007 provided a unique opportunity to evaluate the effectiveness of control measures, including movement restriction and vaccination. The outbreak was initially tackled by imposing risk-based zones of movement restriction with vaccination introduced later. It is, therefore, difficult to assess independently the contribution of vaccination alone to controlling the outbreak as it may already largely have ‘burned out’ in the closed population caused by movement restriction 22. Modelling was therefore used to determine whether earlier use of vaccination could realistically have made a substantial contribution to bringing the outbreak under control 23. It was demonstrated that early vaccination used with other disease control measures would indeed have been very effective at containing the Australian outbreak, reducing the clinical and economic impact, even though some animals would have become subclinically infected. Ideally, a 3 km zone of vaccination around infected premises would be used, but a 1 km zone would be effective. The recombinant canarypox-vectored vaccine used was consistent with disease eradication because differentiation of infected from vaccinated animals (the so-called DIVA strategy) using serological assays was possible with this HA only based vaccine.

Increasingly, infectious disease models are incorporating human factors that may influence how disease spreads and the outcome of disease control measures 24. In modelling the effectiveness of ring vaccination in containing the Australian influenza outbreak, Garner *et al*. 23 assumed compliance with imposed disease control measures and reporting of cases. Contact networks determine how well mixed populations are, for example the use of shared training grounds, such as those seen in Newmarket, UK, can bring horses from different yards into contact in moving from training yards along shared horse walks. Using information about the underlying contact network structure of premises involved in the Australian outbreak as well as taking the geography of the area into account (i.e. information on the location of premises relative to one another) provided the best description of the early spread of the epidemic 25.

## What remains to be done?

As mentioned above, a major limitation of mathematical models is that they are only as good as the available data used to inform them. Where data are lacking, assumptions have to be made. Current gaps include a lack of data that would allow estimation of the effective reproductive number in vaccinated animals. More extensive studies may reveal a graded impact of antibody levels on infectiousness rather than animals simply becoming infectious or not at a certain threshold value of antibody level. There are limitations of the assays used to measure the equine immune response, with most studies limited to assessing the contribution of antibody to protection and assays often using correlates of protection rather than directly measuring neutralisation of the virus. Furthermore, newer vaccine technologies that promote cell-mediated immunity and the potential for mixed vaccination histories as new vaccines are adopted adds further complexity to the immunological landscape of the host. There is also a dynamic interaction between the host and the virus; mutations in the virus are selected for by the presence of host antibodies. Therefore, high levels of vaccine-induced antibody in a population may drive changes in the virus that ultimately lead to vaccine breakdown and an increased likelihood of an outbreak occurring. This review has focused on models to study the transmission of virus between hosts, but models examining the dynamics of the interactions occurring within hosts are increasingly coming to the fore (e.g. 26–27). Most of the studies described here are static or only consider the risk of outbreaks occurring over a relatively short time frame. Mathematical models have yet to disentangle fully the different influences vaccination has on the latent and infectious periods, viral loads in individuals that do become infected and transmission rates. Technologically advanced methods to analyse changes in the viral genome during epidemics are becoming more affordable; the availability of these kinds of data for inclusion in mathematical models 28 will allow more reliable inferences to be made about disease transmission and control.

## Conclusions

Mathematical modelling studies do not replace epidemiological studies and animal experiments, but augment them. In many instances, modelling may simply seem to provide confirmation of the obvious, but it is nonetheless important that decisions about vaccination regimens, for example, are based on evidence rather than supposition (Table 1). Modelling has already revealed that some assumptions about how outbreaks progress can be incorrect. The requirement for accurate data to inform models leads to available data being critically reviewed, which may reveal findings that might otherwise have been overlooked and can inform the design of new studies. Finally, modelling has particular value in predicting the likely outcome at the population level of implementing different control measures and is increasingly considered an important tool for development of disease prevention and control measures. However, as outlined in this review, the development of mathematical models to describe a disease such as equine influenza is an iterative process 29. Conclusions drawn from mathematical models must always be critically evaluated; there may be limited data available to test the performance of a model and preconceived ideas may lead to conceptual errors in the construction of a model.

## Supporting information

**Summary in Chinese.**
