# Supplementary material for: What can mathematical models bring to the control of equine influenza?
Source: Equine Vet J. 2013 Aug 2;45(6):784–8. doi: 10.1111/evj.12104 (PMC3935405; doi:10.1111/evj.12104)
Supplement: Supplementary file 1 [file evj0045-0784-Sd1.docx]

*12104*

**数学模型如何能够控制马流感？**

**摘要** 数学模型在传染病的预防控制环节中的地位变得越来越重要。本文将过去10年里与马流感相关的第一线的各种模型研究资料的主要内容作一总结。

**关键词**  马；流感；数学模型；免疫接种
